# Effects of psychology teachers’ didactic performance on student didactic performance

**DOI:** 10.3389/fpsyg.2025.1607024

**Published:** 2025-07-14

**Authors:** Walter Capa-Luque, Aldo Bazán-Ramírez, Luz Elizabeth Mayorga-Falcón, Evelyn Barboza-Navarro, Edmundo Hervias-Guerra, William Montgomery-Urday, Catalina Bello-Vidal, Danna Rocio García-Ramírez

**Affiliations:** ^1^Facultad de Psicología, Universidad Nacional Federico Villarreal, Lima, Peru; ^2^Departamento de Educación y Humanidades, Universidad Nacional José María Arguedas, Andahuaylas, Peru; ^3^Facultad de Psicología, Universidad Nacional Mayor de San Marcos, Lima, Peru; ^4^Facultad de Medicina, Universidad Nacional Federico Villarreal, Lima, Peru

**Keywords:** didactic performance, mediation, teaching, learning, formative assessment

## Abstract

There is an abundance of literature on students’ evaluations of their teachers’ didactic performance. However, few studies have investigated the relationship between teachers’ didactic performance and its effect on students’ didactic performance, with self-reports that have a solid basis in the substantive theory of such measurements. This study presents a cross-sectional and predictive analysis of the effects of teacher didactic performance (TDP) on student didactic performance (DPPS), examined through structural models that incorporate primary, second-order, and mediating factors within a causal framework. A total of 757 psychology students from a Peruvian public university (171 males and 586 females), selected by non-probabilistic sampling, participated in the study. The scales assessing student perception of teaching didactic performance and self-assessment of their didactic performance were administered. The structural regression model analyzing the direct and indirect effects of the six TDP criteria on the six DPPS criteria presented satisfactory fit indices: χ2(1048) = 2569.701, CFI = 0.928, TLI = 0.923, RMSEA = 0.044, SRMR = 0.064. This model demonstrates that the indirect effects of teaching performance, mediated by SDP criteria such as pre-current learning, illustration-participation, relevant practice, and feedback-enhancement, have a joint impact of 77% on the student criterion evaluation-application (transfer of disciplinary competencies). The second structural model analyzing the direct and indirect effects of the two second-order factors (teaching and formative assessment) of the TDP on the six criteria of the DPPS also presented adequate fit indices: χ2(1061) = 2564.619, CFI = 0.929, TLI = 0.924, RMSEA = 0.043, SRMR = 0.058. Together, the two second-order factors presented indirect effects with an overall impact of 69% on the criterion evaluation-application. Finally, the third model, which incorporates two chain mediators, analyzes the effects of the two second-order quantitative factors of the TDP on the two second-order quantitative factors of the DPPS. This model highlights that the highest ranking indirect effect between teaching and student criterion improvement-application occurs when formative assessment serves as a mediator. It is concluded that the possibility for students to improve, apply, and transfer their professional competencies to the solution of disciplinary problems depends on the optimization of formative assessment, which is linked to the teaching factor that corresponds to the teacher’s didactic performance.

## 1 Introduction

Psychoeducational processes, particularly those related to teaching and learning in university education, involve teacher–student interactions, with planned and intentional actions of the teacher to promote, regulate, and enhance the acquisition of academic-professional skills and competencies, as well as skills and values for sustainable development in students (Morales et al., 2017; Silva et al., 2014). These teaching behaviors during teaching and learning processes in higher education contexts involve skills and competencies in the field of teaching practice in some disciplines. They are forms of teacher behavior in teaching situations; that is, they are actions deployed by the teacher during didactic interaction to promote learning in their students (Bazán-Ramírez et al., 2022a; Kaplan and Owings, 2001; Mou et al., 2022).

These actions are part of the teacher’s instructional and assessment practices during the learning processes in a particular discipline (Bell et al., 2012; Serbati et al., 2020; Liu and Cohen, 2021; Mou et al., 2022). Such teaching practices are susceptible to evaluation from the perspectives of the student body, educational managers and administrators, educational research itself, and also from the self-evaluation of the teachers themselves. This evaluative practice, employing various methods and techniques, is commonly referred to as teacher performance evaluation.

Didactic performance is a term that describes the synchronous and asynchronous relationships between teachers and students during disciplinary and professional training in practical or theoretical-practical settings. According to Dees et al. (2007), teaching and learning are complex human encounters characterized by a teacher-student dialogue, in which the teacher’s role is crucial in creating an environment conducive to learning. This teacher-student encounter constitutes interactions, which, according to Prince et al. (2020), involve the didactic (teaching) actions of the teacher and the participation and reflection of the students (student actions). These didactic interactions encompass teaching performances (the teacher’s didactic performance) and learning performances (the student’s didactic performance) (Bazán-Ramírez et al., 2022a; Bazán-Ramírez et al., 2023). The teacher’s didactic performance and the student’s didactic performance are functionally related.

In the case of Spain, Gómez and Rumbo (2023) have referred to the *Program to Support the Evaluation of the Teaching Activity of University Teaching Staff*, known as DOCENTIA, which assesses the following dimensions of teacher performance in teaching: teaching planning, teaching development, and associated results. Similarly, teaching dedication is considered transversally and is a precondition for evaluation. On the other hand, based on the conception of 19 university teachers in Portugal, Grácio et al. (2023) identified five dimensions (competencies) of university teacher performance: pedagogy and didactics, multiplicity of knowledge, relational aspects, position concerning knowledge and teaching, and technical and scientific qualification.

In a recent systematic review study on the teaching competencies of university teachers, Moreira et al. (2023) analyzed 51 scientific articles published between 2009 and 2019 and identified three domains of instructional performance: didactic competence, communicative competence, and digital competence. Taking only didactic competence, they identified six domains or competencies: (1) ability to use different active teaching methods, strategies, or techniques to support student learning; (2) ability to structure and manage the course or lessons; (3) ability to implement fair and motivating learning assessment; (4) ability to create a supportive classroom climate and manage the classroom effectively; (5) coaching and mentoring ability; and (6) possess strong content knowledge.

Given the diversity of theoretical approaches to define and characterize didactic performance, the present study based on the perspective interbehavioral of psychology (Ibáñez and Ribes, 2001; Irigoyen et al., 2011; Kantor, 1975; Morales et al., 2013; Silva et al., 2014) postulates that the *didactic performance of the teacher* refers to the behaviors and practices deployed in the didactic interaction to sponsor, regulate and mediate student learning. The teacher is the one who establishes the teaching-learning conditions, structures the teaching and learning content and activities, establishes the achievement criteria to be met by their students, and mediates the students’ relationship with the learning object (Silva et al., 2014). These models, which have been put to the test, have made it possible to assess six dimensions of teacher didactic performance as perceived by students: competence exploration, explicitness of criteria, illustration, supervision of practices, feedback, and evaluation (Bazán-Ramírez et al., 2021).

Student *didactic performance* is conceived as the actions exercised by students in response to the demands of pedagogical interaction, aiming to develop or evoke behaviors, skills, and competencies in accordance with the criteria established by the teacher (Morales et al., 2017). Some models of student didactic performance, including self-assessment, have been tested in undergraduate psychology and biology (Bazán-Ramírez et al., 2023) and with graduate students in educational sciences (Bazán-Ramírez et al., 2022b). These studies have confirmed six dimensions of student didactic performance: precurrent to learning, identification of criteria, illustration-participation, pertinent practice, feedback-improvement, and evaluation-application.

### 1.1 Problem and justification of the study

When there are no studies that allow directly identifying the functional relationships between the dimensions of didactic performance of the teacher and the dimensions of didactic performance of the students during didactic interactions, for example, as reported by Velarde-Corrales and Bazán-Ramírez (2019), the study with self-reports can allow having an approximation of what happens in didactic interactions, regarding the actions of teachers and students. In interactive teaching-learning relationships, although students’ performance corresponds functionally with the didactic performance of the teacher (Bazán-Ramírez et al., 2022a; Bazán-Ramírez et al., 2023), it is also important to highlight that the teacher’s performance precedes the deployment of behaviors by students because the functions of directing, managing and facilitating learning by the teacher correspond to various dispositional factors, such as teaching competencies to design an instructional plan and use technological resources (Morales et al., 2017; Silva et al., 2014).

Although several studies have been reported on student ratings of teaching performance, there are very few studies based on a substantive theory that allow testing explanatory models based on latent variables to understand the variability of the effect of teacher teaching performance criteria perceived by students on student self-rated teaching performance.

In correlation with the above, the present study aims to fill the existing knowledge gap, offering an answer to the following research question: How do the criteria of teacher didactic performance, as perceived by students, affect the psychology of students’ self-evaluated didactic performance in a public Peruvian university?

This research seeks to contribute to the analysis of the mastery of competencies and deficiencies in the didactic performance of teachers and students, to strengthen such competencies through training programs aimed at promoting reflection or self-evaluation on the assessment of the performance criteria used in their classes, leading them to make changes and improve their learning strategies.

In accordance with the research problem formulated, the planned research objectives were: (1) to assess validity based on the internal structure of the construct and the reliability of the instruments. (2) To describe the didactic performance of psychology teachers and students. (3) To evaluate the effect of teacher didactic performance criteria on the didactic performance of psychology students at a public university, as perceived by the student body. (4) To evaluate the indirect effect of the second-order latent factors of teacher performance, “teaching and formative assessment,” on the latent factor of student performance, “assessment-application.” (5) To evaluate the indirect (mediational) effects of the second-order quantitative factors “formative assessment and learning” on the relationship between teaching (teaching performance competency factor), and assessment-application (student performance competency factor).

## 2 Materials and methods

### 2.1 Design and variables

**Figure 1:**
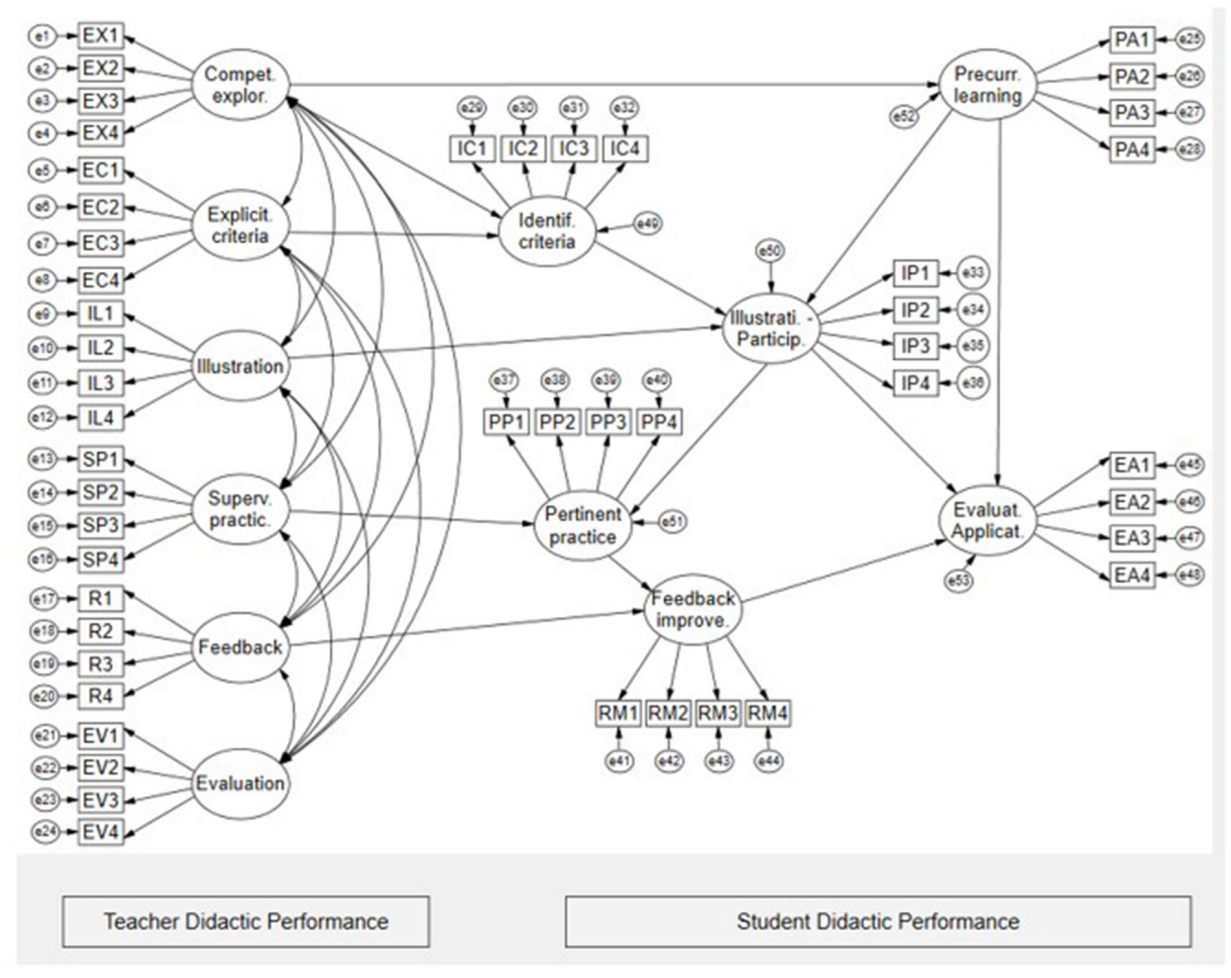
Model of direct effects and indirect on didactic performance in university students.

The study employs a non-experimental and cross-sectional design, as it is multivariate and predictive of the relationships (Hair et al., 2008). The model proposed as the central hypothesis of the study is that teacher didactic performance criteria have direct and indirect effects on student didactic performance criteria (see Figure 1).

#### 2.1.1 Variables of the didactic performance of the teacher as perceived by students

##### 2.1.1.1 Competence exploration

The teacher evaluates the aptitudes, knowledge, and prior skills of their students to determine how well they can learn a specific teaching topic.

##### 2.1.1.2 Explicitness of criteria

The teacher clearly states the expected achievement criteria for student learning and explains how these criteria are to be met.

##### 2.1.1.3 Illustration

The teacher illustrates to the students how the activities are developed, explains what they consist of, and what is required to solve a particular problem within the framework of professional work.

##### 2.1.1.4 Supervision of practices

The teacher monitors the work of his students and accompanies them in solving problems.

##### 2.1.1.5 Feedback

Feedback to the learner regarding the achievement criteria that are implicit in their own learning process corrects and supports them in meeting the achievement criteria.

##### 2.1.1.6 Evaluation

Contrasting the learner’s actual level of performance against a performance considered in the teacher’s initial formulation as a learning objective.

#### 2.1.2 Self-perceived student teaching performance variables

##### 2.1.2.1 Precurrent to learning

The student body demonstrates the knowledge and competencies necessary for the beginning of a subject or topic.

##### 2.1.2.2 Identification of criteria

The student identifies and explains what the achievement criteria made explicit by the teacher are and how they must be satisfied.

##### 2.1.2.3 Illustration-participation

Solves problems as a professional would in his or her discipline, participating in classes in accordance with the structure learned in class.

##### 2.1.2.4 Pertinent practice

Comply with the activities planned and established in the respective learning unit under the guidance and supervision of the educator.

##### 2.1.2.5 Feedback-improvement

Describe in class what was done, how it was done, and in what context, recognizing successes and mistakes in order to correct the latter, according to the various criteria specified by the teacher.

##### 2.1.2.6 Evaluation-application

Demonstrate solvency with respect to the theoretical-conceptual mastery of the topic addressed in relation to knowledge and competencies in accordance with the expected achievements and their compliance criteria.

### 2.2 Population and sample

The population consists of psychology students enrolled in the year 2024, between their first and sixth years of study, at a public university in Lima. It comprises students between 18 and 30 years of age of both sexes.

The sample comprised 757 students (171 males and 586 females). The sample size was estimated using the structural equation modeling calculator (Soper, 2024) for an effect size of 0.20, a statistical power of 0.90, and a 95% confidence level (*α* = 0.05). The restrictions on access to population resources and the lack of a sampling frame made randomized sampling unfeasible, which is why non-probability convenience sampling was employed.

A total of 72 teachers were evaluated, comprising 38 women. With respect to their academic degree, they were 3 bachelors, 38 masters, and 31 doctors. In relation to the teaching category, there were 37 assistants, 21 associates, and 14 principals.

Inclusion criteria: acceptance of informed consent.

Exclusion criteria: students with medical or psychological treatment.

### 2.3 Instruments

#### 2.3.1 Scale of student perception of the didactic performance of the teachers (EDDO)

The scale reported and validated by Bazán et al. with Peruvian students of educational sciences and students of biological sciences was used (Bazán-Ramírez et al., 2022b; Bazán-Ramírez et al., 2023). This scale aims to identify the didactic interaction performances deployed by the teacher, as perceived by the students, in terms of patterns or frequencies of occurrence, with the goal of enhancing the teacher’s didactic practice and ultimately improving the teaching-learning process.

This self-report consists of 24 items, distributed in six factors (competence exploration, explicitness of criteria, illustration, supervision of practices, feedback, and evaluation) with four response options (never, almost never, almost always, and always).

In a sample of 552 biology students from a public university in Peru, satisfactory evidence of validity based on the internal structure of the construct has been reported (CFI and TLI > 0.95, RMSEA and SRMR < 0.06) as well as internal consistency coefficients with McDonald’s omega between 0.84 and 0.91 (Bazán-Ramírez et al., 2023).

In the present study, the psychometric properties of the EDDO scale were reviewed in the sample of psychology students. Table 1 presents the results of Confirmatory Factor Analysis (CFA) to examine the evidence of validity based on the internal structure of the construct. In this regard, the multifactorial model presented very good fit indices (χ2 = 763, gl = 237, *p* < 0.001, CFI = 0.963, TLI = 0.957, SRMR = 0.029, MRSEA = 0.059 [0.05, 0.06]). The evaluation of the parameters denotes high factor loadings (≥ 0.69). Finally, the Interfactor Covariances ranged from 0.78 to 0.94. Regarding reliability, both Cronbach’s alpha and ordinal omega yielded coefficients of 0.98, indicating high precision in the scores estimated by the scale.

**Table 1:** Confirmatory factor analysis and internal consistency reliability of the EDDO.

Factor	Items	Estimator	SE	Z	*p*	λ standard
Factor 1 Competence exploration	DD1	0.69	0.03	20.2	< 0.001	0.72
	DD2	0.75	0.03	26.5	< 0.001	0.86
	DD3	0.76	0.03	25.9	< 0.001	0.85
	DD4	0.63	0.03	23.3	< 0.001	0.79
Factor 2 Explicitness of criteria	DD5	0.71	0.03	22.9	< 0.001	0.78
	DD6	0.69	0.03	25.8	< 0.001	0.84
	DD7	0.74	0.03	27.2	< 0.001	0.87
	DD8	0.77	0.03	27.1	< 0.001	0.87
Factor 3 Illustration	DD9	0.67	0.03	25.1	< 0.001	0.83
	DD10	0.73	0.03	26.5	< 0.001	0.86
	DD11	0.65	0.03	19.4	< 0.001	0.69
	DD12	0.76	0.03	26.4	< 0.001	0.85
Factor 4 Supervision of practices	DD13	0.80	0.03	28.1	< 0.001	0.88
	DD14	0.81	0.03	29.2	< 0.001	0.90
	DD15	0.75	0.03	27.5	< 0.001	0.87
	DD16	0.76	0.03	27.8	< 0.001	0.88
Factor 5 Feedback	DD17	0.72	0.03	26.9	< 0.001	0.86
	DD18	0.80	0.03	27.7	< 0.001	0.88
	DD19	0.78	0.03	27.9	< 0.001	0.88
	DD20	0.70	0.03	24.6	< 0.001	0.81
Factor 6 Evaluation	DD21	0.73	0.03	23.3	< 0.001	0.79
	DD22	0.79	0.03	26.9	< 0.001	0.87
	DD23	0.74	0.03	24.9	< 0.001	0.83
	DD24	0.69	0.03	24.1	< 0.001	0.81

	F1	F2	F3	F4	F5	F6
F1	1.00					
F2	0.82	1.00				
F3	0.80	0.94	1.00			
F4	0.78	0.90	0.92	1.00		
F5	0.80	0.89	0.92	0.94	1.00	
F6	0.84	0.86	0.87	0.85	0.90	1.00
Alpha	0.916	0.939	0.919	0.934	0.950	0.926
Omega	0.929	0.959	0.934	0.935	0.959	0.928
Scale	Ordinal α = 0.983		ω ordinal = 0.987			

SE = standard error, Z = Z score, *p* = significance, λ standard = standardized factor loading.

#### 2.3.2 Self-Assessment Scale of Student Didactic Performance (EADDE)

Self-Assessment Scale of Student Didactic Performance (EADDE) was validated with Peruvian students from educational sciences and biological sciences (Bazán-Ramírez et al., 2022b; Bazán-Ramírez et al., 2023). The scale comprises 24 items organized into six subscales or dimensions of student didactic performance: precurrent to learning, identification of criteria, illustration-participation, pertinent practice, feedback-improvement, and evaluation-application. Each subscale presents four items with graded responses (never, sometimes, almost always, always). In a study by Bazán-Ramírez et al. (2023) involving biology students from a Peruvian university, the scale demonstrated adequate construct validity (CFI and TLI ≥ 0.95, RMSEA and SRMR < 0.08) and reliable factor scores (ω between 0.88 and 0.93).

For use in the present study, the psychometric properties of the EADDE scale were examined in the sample of psychology students (see Table 2).

**Table 2:** Confirmatory factor analysis and internal consistency reliability of the EADDE.

Factor	Items	Estimator	SE	Z	*p*	λ Standard
Factor 1 Precurrent to learning	DE1	0.60	0.03	21.00	< 0.001	0.74
	DE2	0.71	0.03	27.00	< 0.001	0.88
	DE3	0.63	0.03	23.30	< 0.001	0.80
	DE4	0.64	0.03	23.50	< 0.001	0.80
Factor 2 Identification of criteria	DE5	0.54	0.03	17.50	< 0.001	0.66
	DE6	0.51	0.02	20.30	< 0.001	0.73
	DE7	0.54	0.02	22.00	< 0.001	0.77
	DE8	0.63	0.03	24.50	< 0.001	0.83
Factor 3 Illustration—participation	DE9	0.52	0.03	19.60	< 0.001	0.69
	DE10	0.58	0.03	21.70	< 0.001	0.75
	DE11	0.52	0.03	15.00	< 0.001	0.56
	DE12	0.55	0.03	20.40	< 0.001	0.72
Factor 4 Pertinent practice	DE13	0.73	0.03	25.30	< 0.001	0.84
	DE14	0.75	0.03	25.10	< 0.001	0.83
	DE15	0.68	0.03	26.30	< 0.001	0.86
	DE16	0.61	0.03	23.00	< 0.001	0.79
Factor 5 Feedback-improvement	DE17	0.64	0.03	25.20	< 0.001	0.84
	DE18	0.64	0.03	24.70	< 0.001	0.82
	DE19	0.65	0.03	24.00	< 0.001	0.81
	DE20	0.58	0.03	21.90	< 0.001	0.76
Factor 6 Evaluation—application	DE21	0.60	0.03	23.30	< 0.001	0.81
	DE22	0.61	0.03	21.60	< 0.001	0.77
	DE23	0.57	0.03	20.50	< 0.001	0.74
	DE24	0.51	0.03	18.80	< 0.001	0.69

	F1	F2	F3	F4	F5	F6
F1	1.00					
F2	0.59	1.00				
F3	0.61	0.97	1.00			
F4	0.54	0.80	0.92	1.00		
F5	0.50	0.80	0.91	0.87	1.00	
F6	0.66	0.71	0.82	0.68	0.71	1.00
Alpha	0.917	0.878	0.833	0.895	0.923	0.886
Omega	0.930	0.911	0.858	0.896	0.941	0.912
Scale	ordinal α = 0.966		ordinal ω = 0.975			

SE = standard error, Z = Z score, *p* = significance, λ Standard = standardized factor loading.

According to the global evaluation indices, the factorial model of the EADDE presents an adequate fit (χ2 = 842, gl = 237, *p* < 0.001, CFI = 0.939, TLI = 0.929, SRMR = 0.044, MRSEA = 0.063 [0.05, 0.07]), that is, the internal structure of the construct is reproduced according to what is established by the theory. Table 2 also shows that the factors contain items that saturate with high factor loadings. As for the Interfactor Covariances, these vary between moderate and strong. With respect to reliability, the scale, in general, as well as its dimensions, presents ordinal alpha and omega values that denote high precision in the measured scores.

### 2.4 Procedure

The instruments were administered both in person and through the virtual modality with a form designed in Google Drive. In the case of the virtual applications, the URL links were sent to the study population’s email addresses and WhatsApp accounts. At the beginning of the form, an informative text was presented outlining the research objectives, potential benefits and risks, and the request for informed consent. In the face-to-face application, consent was printed and signed in person. For the virtual application, consent was obtained online and signed virtually.

The data were collected anonymously, treated with confidentiality, coded, and entered into a database. The data collection period was between July and 1 August 30, 2023.

### 2.5 Data analysis

In the first phase, the psychometric properties of the research instruments were reviewed with the study sample.

SPSS version 27 was used for descriptive and comparative analyses. Both psychometric analyses and structural regression model analyses to examine the direct and indirect effects of teacher didactic performance on students’ didactic performance criteria used the freely distributed software R version 4.3.1 and RStudio version 2023.06.2.

The two SEM models were processed using the robust maximum likelihood estimation (MLM) method. As in SEM models, the evaluation of structural parameters (interfactor relationships and standard errors), in addition to fit indices, is of primary interest. Robust ML was used as it is more advantageous than the WLSMV estimator (suggested for ordinal data) when the sample is less than 1,000 cases (Li, 2016). Even when instruments have two to four response categories, robust ML offers unbiased estimates of factorial correlations (Rhemtulla et al., 2012). The recommended robust fit indices were used to assess the overall fit of the structural models (Hu and Bentler, 1999; Keith, 2019). The Comparative Fit Index (CFI) and the Tucker-Lewis index (TLI) denote adequate fit when their values are ≥ 0.90 and good fit if they are ≥ 0.95; the Root Mean Square Error of Approximation (RMSEA) denotes adequate fit when their values are ≤ 0.08 and good fit if their values are ≤ 0.05; and the Standardized Root Mean Square Residual (SRMR) denotes adequate fit when their values are ≤ 0.08 and good fit if they are ≤ 0.06.

Mediation analyses were performed between second-order factors, which served as metric variables for both teacher teaching performance as perceived by students and student self-assessment of teaching performance, using the PROCESS macro module version 4.2 for SPSS (Hayes, 2022). The results of this analysis were based on bootstrap resampling of 5,000 cases. According to Hayes (2022), Confidence Intervals (CIs) are significant when they do not contain zero. In the case of mediational models, confidence intervals (CIs) based on bootstrapping enable us to identify indirect effects and determine, among significant mediations, the order of superiority.

Both structural regression models of latent variables, which are part of structural equation modeling (SEM), and mediation analysis with quantitative variables are techniques for analyzing direct and indirect effects in the relationships between variables. According to Keith (2019), in cross-sectional studies, these techniques correspond to weak causality relationships. In this case, weak causality relationships are configured when three conditions are met (Byrne, 2010; Keith, 2019): (1) the existence of a functional relationship between variables, (2) the cause precedes the effect in a real or logical way in time and (3) the relationship is not spurious. It is worth noting that strong causality is typically associated with experimental studies.

## 3 Results

### 3.1 Students’ perception of the didactic performance of teachers

Table 3 shows the students’ perception of their teachers’ didactic performance in the six competency criteria.

**Table 3:** Descriptive analysis of the teacher’s didactic performance for each criterion according to the student body.

*n* = 632	Minimum	Maximum	Mean [95% CI]	SD	CV	Z
Competence exploration	0	12	7.58 [7.34, 7.81]	3.01	0.396	−0.250
Explicitness of criteria	0	12	8.66 [8.43, 8.90]	3.06	0.354	0.101
Illustration	0	12	8.54 [8.30, 8.77]	2.99	0.350	0.060
Supervision of practice	0	12	8.68 [8.43, 8.93]	3.23	0.372	0.105
Feedback	0	12	8.72 [8.48, 8.97]	3.14	0.359	0.120
Evaluation	0	12	7.94 [7.69, 8.18]	3.11	0.392	−0.135

SD = Standard Deviation, CI = Confidence Interval, CV = coefficient of variation, Z = Z score, *n* = sample.

According to the confidence intervals of the mean and Z-scores, the criteria rated as the best performers are feedback, supervision of practice and learning activities, explicitness of criteria, and illustration. On the other hand, the criteria for competency requiring optimization are competence exploration and evaluation.

### 3.2 Students’ self-assessment of their teaching performance

According to the Z scores and confidence intervals of the mean shown in Table 4, the competence performance criteria that the students best developed are, on the other hand, feedback-improvement, pertinent practice, and identification of criteria. Performance that needs to be corrected and strengthened includes competency criteria pre-current to learning, illustration-participation, and evaluation-application.

**Table 4:** Descriptive analysis of self-assessment of their teaching performance students for each criterion.

*n* = 632	Minimum	Maximum	Mean [95%CI]	SD	CV	Z
Precursors to learning	0	12	6.84 [6.63, 7.06]	2.739	0.400	−0.513
Identification of criteria	0	12	8.56 [8.37, 8.75]	2.416	0.282	0.141
Illustration—participation	0	12	8.12 [7.92, 8.31]	2.481	0.306	−0.027
Pertinent practice	0	12	8.61 [8.39, 8.84]	2.912	0.338	0.162
Feedback—improvement	0	12	8.91 [8.70, 9.12]	2.664	0.299	0.275
Evaluation—application	0	12	8.09 [7.90, 8.29]	2.505	0.310	−0.037

SD = Standard Deviation, CI = Confidence Interval, CV = coefficient of variation, Z = Z score, *n* = sample.

### 3.3 Effect of teacher didactic performance criteria on the didactic performance of psychology students at a public university

**Figure 2:**
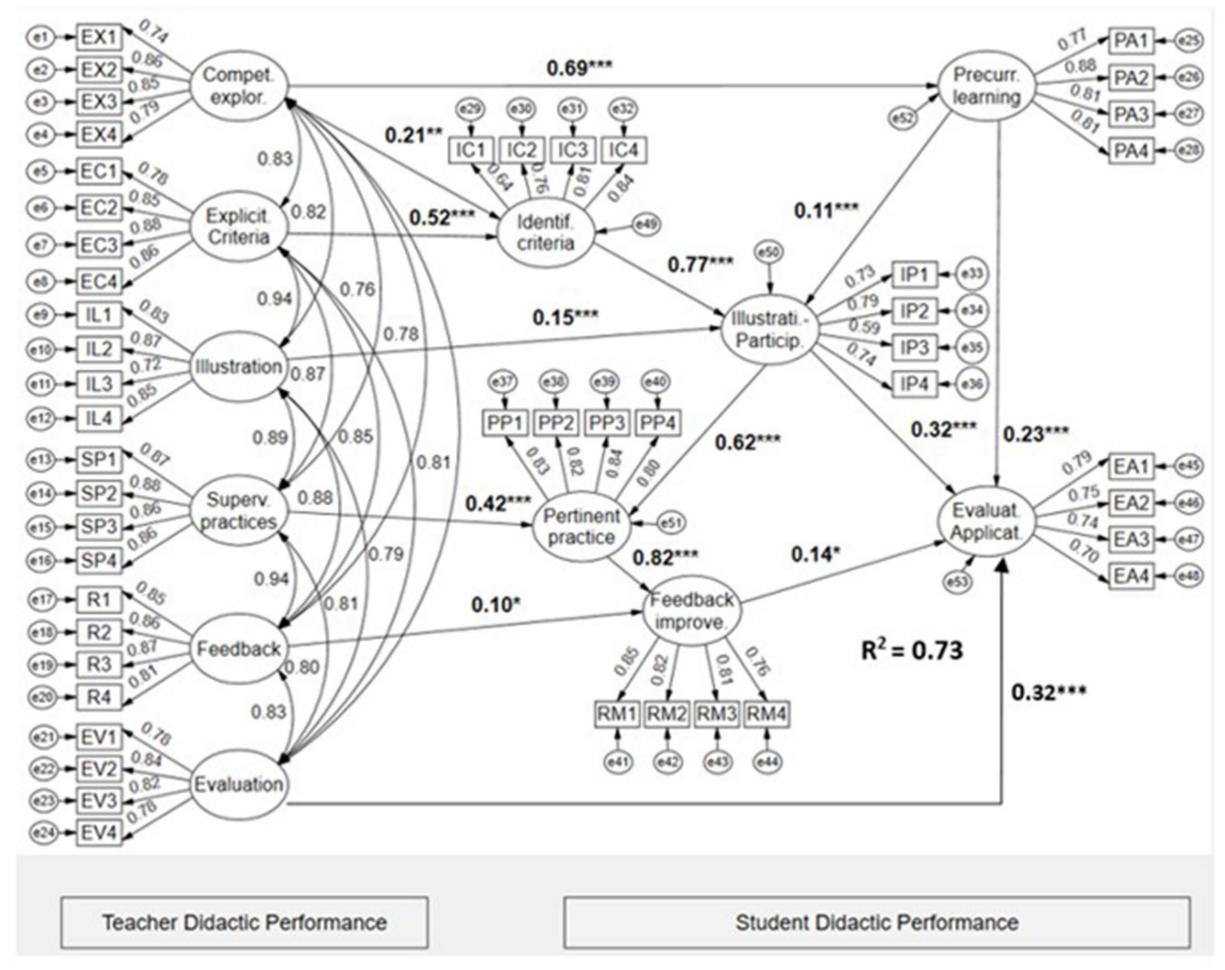
Latent structural regression model of the effects of didactic teaching performance on the didactic performance of psychology students. The values (bold) shown in the Figure are the structural regression coefficients. * *p* < 0.05, ** *p* < 0.01, *** *p* < 0.001.

Figure 2 presents the results of the structural regression analysis, which tests the proposed hypothesis that the teacher’s didactic performance criteria have both direct and indirect effects on the student’s didactic performance criteria. The structural model presents satisfactory fit indices: χ2(1051) = 2400.336, CFI = 0.936, TLI = 0.932, RMSEA = 0.041 [0.039, 0.043], SRMR = 0.055. Therefore, the data support the validity of the structural model.

It can be seen in the model resulting (Figure 2) that competency exploration by the teacher has a direct effect of 47% on the actions pre-current learning performed by the students. Likewise, the factors of teaching performance, competence exploration, and explicitness of criteria have a direct impact of 49% on the identification of the criteria by the students. The criterion illustration student participation contains an impact of 87% of the teaching performance criteria illustration (direct effect) and explicitness of criteria (indirect effect), as well as the direct effects of precurrents to learning deployed by students. The fourth performance criterion, pertinent practice, exercised by students presents an R^2^ of 0.90 as a result of the direct effects of teacher supervision of practices and the criterion illustration-participation student. The endogenous feedback-improvement factor corresponding to student performance is affected directly (feedback) and indirectly (supervision of practice and learning activities) by teaching performance criteria (R^2^ = 0.82). Finally, the indirect effects of teaching performance through mediators corresponding to student didactic performance criteria, such as precurrents to learning, illustration-participation, and feedback-improvement, plus the direct effect of the teacher’s evaluation factor, have a joint impact of 73% (total R^2^ of the model) on student evaluation-application criteria.

The latent correlations (Table 5) between teacher teaching performance criteria and student teaching performance criteria are positive and correspond to a large effect size (r > 0.50).

**Table 5:** Matrix of correlations of latent factors of teacher performance and student performance.

	1	2	3	4	5	6	7	8	9	10	11
1_F1_Competece explorat.	1.00										
2_F2_Explicitness of crite.	0.83	1.00									
3_F3_Illustration	0.82	0.96	1.00								
4_F4_Supervision of pract.	0.76	0.87	0.90	1.00							
5_F5_Feedback	0.78	0.85	0.89	0.94	1.00						
6_F6_Evaluation	0.81	0.78	0.82	0.80	0.83	1.00					
7_F1_Precurrent to learn.	**0.69**	**0.57**	**0.57**	**0.52**	**0.54**	**0.56**	1.00				
8_F2_Identification of crit.	**0.65**	**0.70**	**0.67**	**0.62**	**0.61**	**0.58**	0.45	1.00			
9_F3_Illustration-particip.	**0.70**	**0.71**	**0.76**	**0.69**	**0.69**	**0.66**	0.55	0.91	1.00		
10_F4_ Pertinent practice	**0.74**	**0.80**	**0.84**	**0.85**	**0.82**	**0.74**	0.55	0.79	0.88	1.00	
11_F5_Feedback-improv.	**0.65**	**0.72**	**0.75**	**0.80**	**0.80**	**0.71**	0.45	0.51	0.58	0.69	1.00
12_F6_Evaluation-applic.	**0.73**	**0.70**	**0.73**	**0.69**	**0.71**	**0.77**	0.64	0.66	0.75	0.69	0.68

The coefficients in bold are the correlations between teacher performance criteria and student performance criteria.

**Figure 3:**
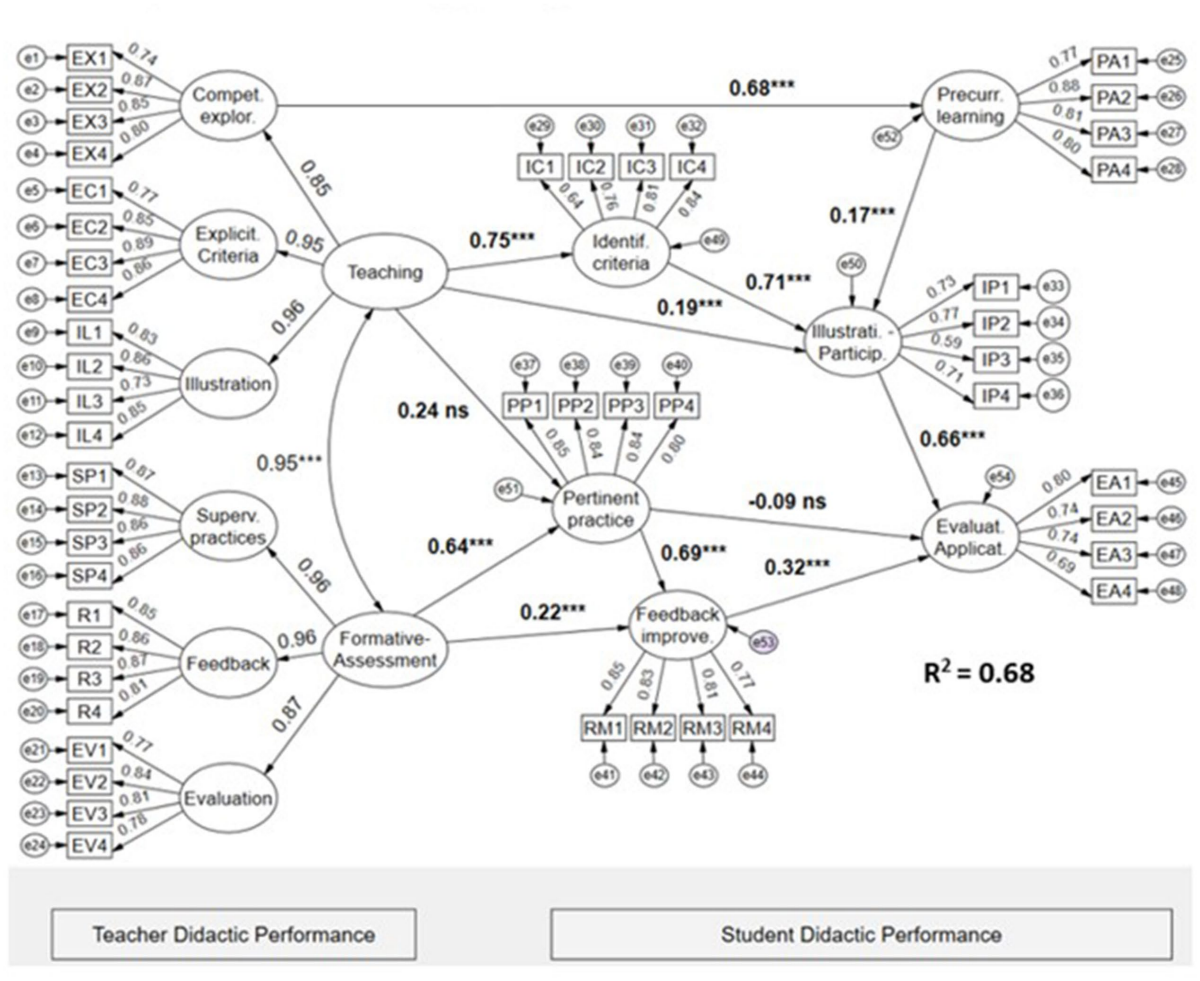
Latent structural regression model of the effects of formative teaching and assessment on didactic performance criteria in psychology students. The values (bold) shown in the Figure are the structural regression coefficients. * *p* < 0.05, ** *p* < 0.01, *** *p* < 0.001, ns = not significant (*p* > 0.05).

The second structural model (see Figure 3) configures the six criteria of teaching performance into two second-order factors (Teaching and Formative Assessment), which are directly and indirectly related to the six criteria of student performance. The examined model presents fit indices teaching satisfactory overall: χ2(1061) = 2757.704, CFI = 0.920, TLI = 0.915, RMSEA = 0.046 [0.044, 0.048], SRMR = 0.064. Therefore, the evidence supports the validity of the model. Figure 3 shows that the most significant indirect effect is between the factor of teaching and evaluation application, mediated through a path involving double serial mediation (identification of criteria and illustration-participation). The other factor in teaching performance, called formative evaluation, channels its indirect effects on evaluation-application through two-chain mediators (pertinent practice and feedback-improvement). In summary, the two second-order factors account for an explained variance of 68% in student teaching performance, specifically in the evaluation-application domain.

### 3.4 Multiple mediation analysis

To determine the indirect effects between the second-order factors of the teacher and the second-order factors of student teaching performance, a third-order model is examined. Thus, Table 6 examines, through regression analysis (Hayes model 6), the impact of the mediating variables formative assessment (M1) and learning (M2) on the relationship between teaching as a teaching competence (X) and the application-transfer competence (Y) in psychology students.

**Table 6:** Multiple mediation analysis with two mediating variables in a causal chain.

Model 1	Outcome variable: formative evaluation						
	R = 0.87, R^2^ = 0.76, MSE = 16.18, F = 2356.49, df1 = 1, df2 = 755, *p* = 0.000						
	Model	Coeff	SE	t	*p*	LLCI	ULCI
	Constant	5.03	0.45	11.04	0.000	4.13	5.92
	Teaching	0.84	0.01	48.54	0.000	0.80	0.87
Model 2	Outcome variable: learning						
	R = 0.75, R^2^ = 0.56, MSE = 19.87, F = 496.28, df1 = 2, df2 = 754, *p* = 0.000						
	Model	Coeff	SE	t	*p*	LLCI	ULCI
	Constant	7.36	0.54	13.52	0.000	6.29	8.42
	Teaching	0.23	0.03	5.90	0.000	0.15	0.30
	Formative evaluation	0.40	0.04	10.10	0.000	0.32	0.48
Model 3	Outcome variable: improvement-application						
	R = 0.89, R^2^ = 0.80, MSE = 10.27, F = 1026.04, df1 = 3, df2 = 753, *p* = 0.000						
	Model	Coeff	SE	t	*p*	LLCI	ULCI
	Constant	2.65	0.43	6.07	0.000	1.79	3.50
	Teaching	−0.08	0.02	−3.12	0.001	−0.14	−0.03
	Formative evaluation	0.46	0.03	15.06	0.000	0.40	0.52
	Learning	0.56	0.02	21.47	0.000	0.51	0.61
Model 4	Total model effect						
	Outcome variable: improvement-application						
	R = 0.73 R^2^ = 0.53 MSE = 24.32 F = 862.81 df1 = 1 df2 = 755 *p* = 0.000						
	Model	Coeff	SE	t	*p*	LLCI	ULCI
	Constant	10.28	0.55	18.41	0.000	9.18	11.38
	Teaching	0.62	0.02	29.37	0.000	0.58	0.66
	Direct effect of X on Y						
		Effect	SE	t	*p*	LLCI	ULCI
		−0.08	0.02	−3.12	0.001	−0.14	−0.03
	Indirect effects of X on Y						
		Effect	BootSE	BootLLCI	BootULCI		
	Total	0.714	0.038	0.641	0.792		
	Ind1	0.391	0.031	0.332	0.455		
	Ind2	0.129	0.027	0.077	0.185		
	Ind3	0.193	0.023	0.148	0.239		
	(C1)	0.262	0.037	0.188	0.336		
	(C2)	0.198	0.042	0.114	0.284		
	(C3)	−0.06	0.046	−0.150	0.028		

R = regression coefficient, R^2^ = coefficient of determination, MSE = Mean Square Error, F = F-Test, df = degrees of freedom, *p* = significance, se = standard error, t = t-Test, LLCI = lower limit of the confidence interval, ULCI = upper limit of the confidence interval, Ind = indirect effect, Boot = bootstrapping, C = contrast, X = independent variable, Y = dependent variable.

The first regression model shows that the teaching variable is statistically significantly effective on the first mediating variable (a_1_ = *B* = 0.84, *p* < 0.001). The second regression model presents results for the second mediating variable (learning). It can be seen that both the teaching variable (a_2_ = *B* = 0.23, *p* < 0.001) and the mediating variable 1 (d_21_ = *B* = 0.40, *p* < 0.001) exert significant effects on learning. The third regression analysis shows that the independent variable (c´ = *B* = −0.08, *p* < 0.01) and the two mediating variables, formative assessment (b_1_ = *B* = 0.46, *p* < 0.001) and learning (b_2_ = *B* = 0.56, *p* < 0.001) exert statistically significant effects on the dependent variable. The fourth analysis reveals that the total effect of the model is mediated (c = *B* = 0.62, *p* < 0.001).

**Figure 4:**
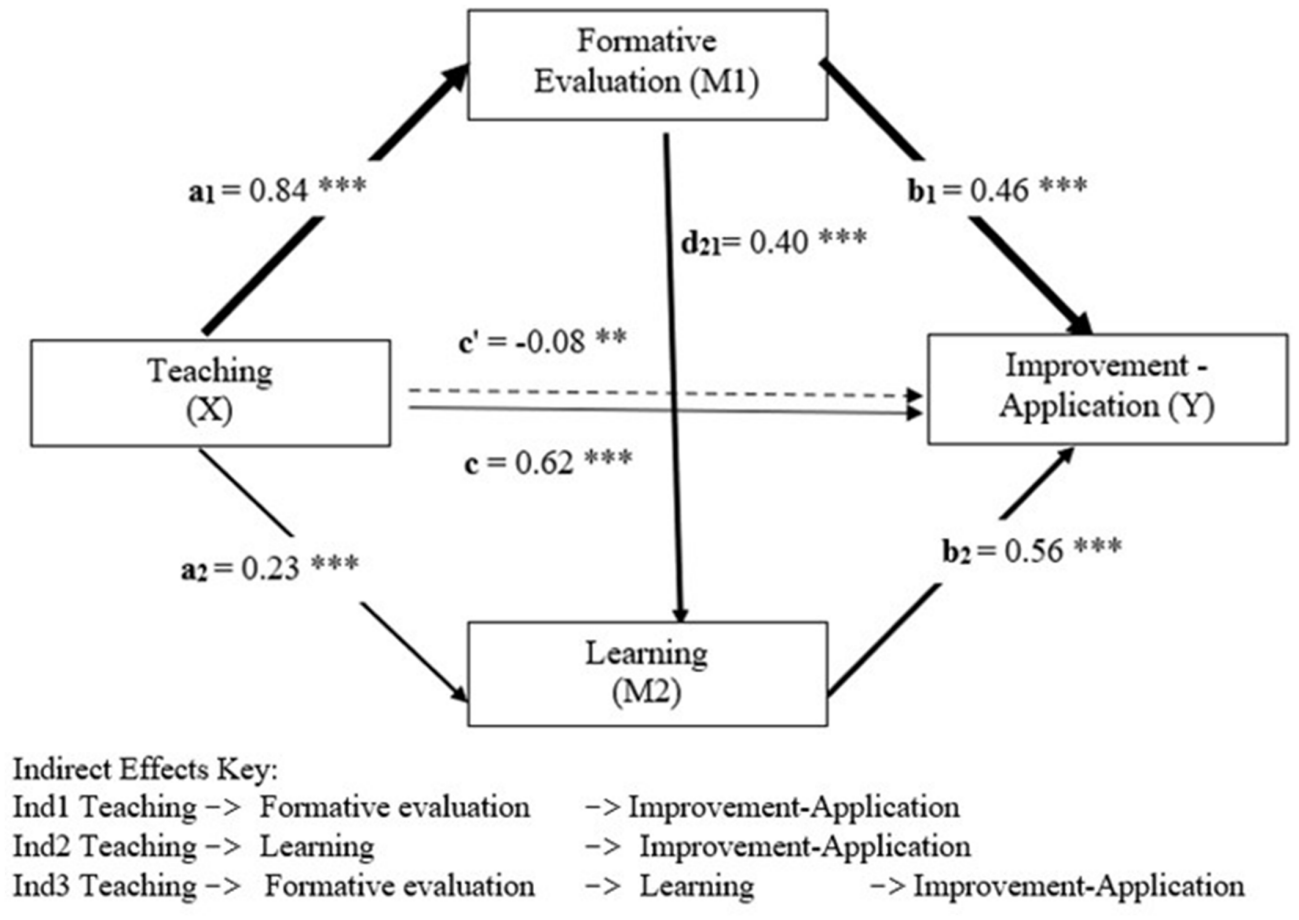
Model of effects mediational in the teaching relationship on application—transfer competence in psychology students. The values shown in the figure are the regression coefficients unstandardized. ** *p* < 0.01 *** *p* < 0.001, c: total effect, c’ direct effect, X = Independent variable, Y = Dependent variable, M1, Mediating variable 1, and M2, Mediating variable 2.

Finally, Table 6 indicates that the three indirect effects are statistically significant, as indicated by the 95% confidence intervals. The contrast results allow us to identify the order of superiority of the indirect effects. In this sense, indirect effect 1, operating on the first mediating variable (see the path of the thickest dates in Figure 4), is superior to the other two indirect effects. The second most significant indirect effect corresponds to the pathway involving both mediating variables in sequence (X → M1 → M2 → Y), as illustrated in Figure 4.

## 4 Discussion

The present study provides updated information on the recent state of the variables analyzed in the academic context of higher education in Peruvian students and describes and explains how they affect or regulate the criteria of the didactic performance of teachers in the academic and scientific training of future psychology professionals.

In the first instance, an important contribution of the study, at the methodological level, was to provide new evidence on the accuracy (reliability) and suitability of the instruments to derive correct interpretations and diagnoses from their scores, for which there was evidence of validity based on the internal structure of the construct obtained. As Muñiz (2018), providing instruments with suitable qualities of validity and reliability will not only allow the development and increase of new research that expand the frontiers of scientific knowledge but also facilitate the work of professionals, who as agents of social change, seek the generation of better quality graduates and who can constitute the efficient workforce.

Our results of the univariate analysis regarding the teachers’ didactic performance highlight feedback, supervision of practice and learning activities, the explicitness of criteria, and illustration as the most efficiently used; this expresses that the students’ perception of these three competency criteria of teachers has significant implications in their educational process. When comparing these results with those of Bazán-Ramírez et al. (2023), there is concordance regarding the explicitness of the criteria, but not with the evaluation criterion.

On the other hand, in terms of the students’ didactic performance competency criteria, feedback-improvement, pertinent practice, and identification of criteria stood out as those with the best development and efficient use. On the other hand, the performance competency criteria that need to be corrected and strengthened are precurrent for learning, illustration-participation, and evaluation-application. These results partially correspond to the study of Bazán-Ramírez et al. (2023), who identified outstanding student performance in undergraduate students of biological sciences as relevant practice and identification of criteria, but not in feedback-improvement.

In accordance with the objectives referring to the effect of the criteria on the didactic performance of the teacher as perceived by the students on the didactic performance of the students, three predictive models were tested. The interpretation of the data is basically supported by the conceptual framework of the study, given that there are no similar investigations with which to contrast the results, with the exception of the only precedent carried out in a sample of graduate students in education (Bazán-Ramírez et al., 2022a) which is close to the present study.

The first model consisted of evaluating through structural regression analysis of first-order latent variables the direct and indirect effects of the six teacher didactic performance criteria on the six student didactic performance criteria. The central finding of this model indicates that the indirect effects of teacher performance mediated by student performance criteria, i.e., precurrent for learning, illustration-participation, and feedback-enhancement, had a joint impact of 73% on the student criterion evaluation-application (transfer of disciplinary competencies). This means that the possibility of transferring the performance parameters to new problems and situations (evaluation-application) depends on the fulfillment of the student didactic performance criteria and the evaluation of the didactic performance of the teacher as perceived by the student body.

Specifically, the model has allowed us to observe that the teacher’s exploration of competencies has a direct effect on the actions of pre-current learning deployed by students. This implies that the teacher, when evaluating the skills and competencies necessary for learning the topic in a class to be started, not only encourages the student to deploy his/her potential capabilities for new learning but also that the student identifies what they need to know and what the didactic situation requires.

It was also identified that the factors of teaching performance, competency exploration, and the explicitness of criteria have a direct impact on students’ identification of criteria. This means that the evaluation of the student’s potential knowledge and capabilities, together with the explicitness of the disciplinary and didactic criteria that the student must satisfy with his/her performance, allows adjusting his/her didactic performance (what he/she will do, how, when and where) and reaching the expected achievement based on the established criteria.

Another important finding concerns the explicitness of criteria on the part of the teacher and the adjustment of the student’s didactic performance based on the identification of these criteria. In this way, the student is able to create and utilize strategies and resources to learn the forms of action expected by the teacher.

It is worth highlighting the direct and significant impact that the teacher’s supervision of practices has on the criterion of pertinent practice exercised by the students. This means that the didactic adjustment of the student in class, based on the learning situations regulated by the teacher (where the supervision and correction are made possible moment by moment of the trainee’s performance), is conducive to the student demonstrating competent performance adjusted to the requirements and achievement criteria.

Finally, it has been found that feedback (enabling the student to get in touch with his own behavior and its possible variants) allows the student to monitor to what extent he is in a position to meet the achievement criterion(s), how close he is, and what adjustments to make to achieve the satisfaction of the expected achievement criterion(s).

In the analysis carried out in the second structural model on the perception of psychology students regarding the didactic performance of the teacher, the results indicate that the route of the most relevant indirect effects is between the teaching and factor evaluation-application through the route that contemplates double mediation in the identification of criteria and illustration-participation. For the competency criterion formative evaluation of teacher performance, the indirect effects are channeled in series through two mediators: pertinent practice and feedback-improvement. Together, the two second-order factors of teacher didactic performance have an overall impact of 68% on the most important terminal criterion of student didactic performance, which is evaluation-application. Ibáñez and Ribes (2001) as well as Kantor (1975), stated that didactic performance depends on the teacher’s ability to facilitate student learning through effective and meaningful interactions. This vision is complemented by Morales et al. (2013, 2017), who note that didactic performance not only implies knowledge transmission but also fosters an environment conducive to learning, where skills, attitudes, and values are integrated. Additionally, Bazán-Ramírez et al. (2022a) when analyzing the teaching-learning conditions in the context of higher education, emphasized the importance of the didactic performance factors of the teacher that mediate the relationship between the student’s didactic performance and the object of learning.

In the third model, the results of the multiple mediation analysis, which considers formative assessment and learning as mediating variables, show that these factors exert indirect effects with different degrees of relevance between teaching and the transfer of disciplinary competencies in students. The fundamental indirect route to optimize and enhance the possibility that students can apply and transfer the acquired knowledge and competencies is through the optimization of formative assessment, a consequence of the teacher’s didactic performance in teaching. In contrast, although the indirect route through the learning mediator shows significant indirect effects between teaching and the possibility of improvement-application of competencies, it is the one that would have the least impact on the formative quality that enables the transfer and application of competencies in solving disciplinary problems.

### 4.1 Implications for practice

The results of the research could be useful in optimizing curricular experiences, both in theory and practice hours, that benefit the teaching-learning process oriented toward the acquisition and strengthening of skills and academic-professional competencies. In university teacher training, the results would also have implications in the following contexts: (1) educational innovation: to reinforce quality education where teacher training at the higher level involves greater active participation of students, as well as to allow teachers to articulate their didactic competencies in teaching actions. (2) feedback: the mechanism involved would facilitate the conscious self-demand that usually accompanies the self-evaluation of the learner’s own abilities according to the teaching framework established by the teacher. (3) tutoring and educational guidance: this guidance procedure could directly influence the student’s academic commitment and performance by facilitating the identification of mediating factors that enhance or limit learning.

The social impact in terms of the training of professionals (a task entrusted by society to universities) is not only local but also has national and international scope. For example, according to the background information reviewed, there is interest in understanding the didactic teacher-student interaction in professional careers in biology (Peru), education, and psychology (Mexico) so that the findings of this study will allow contrasting educational realities and having the possibility of sharing experiences with these institutions to optimize the training quality of professionals, who will be the agents of social and economic change in the country in the near future.

## 5 Limitations

Some of the most important limitations of the study include the use of non-probabilistic sampling, which undermines the external validity of the research, even though a large sample has been taken (two-thirds of the population) to reduce selection biases of the units of analysis, it is recommended that in future studies randomized samples be taken. The fact that the sample corresponds to psychology students and is from a single public university can also be considered a limitation of the study. It is suggested in future studies to take samples from several universities and different professional careers. Likewise, another limitation could lie in the use of self-reports for the measurement of the variables due to a possible social desirability bias committed by the respondents, even though this undesired effect is minimized by valid and reliable scores, as well as by the quality control of extreme data through the multivariate analysis of centroids with the Mahalanobis distance. To strengthen the findings, in addition to replicating the study, it would be interesting to use an observational methodology for measuring the variables (Velarde-Corrales and Bazán-Ramírez, 2019). Despite these limitations, the present study is relevant because there are few studies aimed at offering models to explain the interactions or effects between the criteria or factors corresponding to the didactic performance of teachers and the performance of students in the university context, especially in a sample of psychology students, in this sense, the present study will fill this knowledge gap. This study will provide a basis for future basic and applied studies.

## 6 Conclusion

1. According to the structural regression model with first-order factors, three student performance criteria (illustration-participation, relevant practice, and feedback-improvement) constitute the mediators of indirect effects of the teaching performance criteria on the student’s competence criteria to evaluate and apply solutions to disciplinary problems of the profession.
2. Between the second-order factors of the teacher’s didactic performance (teaching and formative evaluation) and the student’s evaluation-application criterion, there are indirect effects regulated by the participation of two mediators in a causal chain that corresponds to the student performance criteria. The route of the first mediation is teaching → identification of criteria → illustration-participation → evaluation-application, while the route of the second mediation is formative evaluation → relevant practice → feedback improvement → evaluation-application.
3. The mediational analysis of the second-order quantitative factors highlights that the indirect effect of greater hierarchy between teaching as a teaching competence and the student performance criterion improvement-application is where formative assessment participates as a mediator. This means that the possibility for students to improve, apply and transfer their professional competencies to the solution of disciplinary problems depends on the optimization of the formative evaluation that is linked to the teaching factor that corresponds to the didactic performance of the teacher.

## Data Availability

The raw data supporting the conclusions of this article will be made available by the authors, without undue reservation.
